# The efficacy of angiotensin converting enzyme inhibitors versus angiotensin II receptor blockers on insulin resistance in hypertensive patients: A protocol for a systematic review and meta-analysis

**DOI:** 10.1097/MD.0000000000020674

**Published:** 2020-06-12

**Authors:** Jia Yao, Xiayu Gong, Xiaoyan Shi, Simin Fan, Junmin Chen, Qiu Chen

**Affiliations:** School of Clinical Medicine, Chengdu University of Traditional Chinese Medicine, Chengdu, Sichuan Province, P.R. China.

**Keywords:** angiotensin-converting enzyme inhibitors, angiotensin receptor blockers, blood pressure, hypertension, insulin resistance, meta-analysis

## Abstract

**Background:**

Previous studies have shown inconsistent outcomes in the efficacy of angiotensin-converting enzyme inhibitors (ACE inhibitors) and angiotensin receptor blockers (ARBs) on insulin resistance (IR). Hence, we aim to compare the efficacy of ACE inhibitors with ARBs on IR in hypertensive patients.

**Methods:**

Five electronic databases (included The Cochrane Library, MEDLINE, Embase, Web of Science, and Cochrane Central Register of Controlled Trials) will be searched. Randomized controlled trials (RCTs) will be included if they recruited hypertensive participants for assessing the effect of ACE inhibitors on IR versus ARBs. The primary outcome will be IR (using recognized methods such as homeostasis model assessment of insulin resistance), secondary outcomes will be blood pressure, fasting plasma glucose, fasting plasma insulin. Relevant literature search, data extraction, and quality assessment will be performed by 2 researchers independently, and the third researcher will be involved in a discussion for any disagreements. All analyses will be performed based on the Cochrane Handbook for Systematic Reviews of Interventions. Stata 12.0 software will be used for statistical analysis. The effect size of dichotomous data will be measured using the odds ratio (OR), and the effect size of continuous data will be measured using the standardized mean difference. And 95% confidence intervals will be calculated. Heterogeneity will be tested by *χ*^2^-based Cochran Q statistic and I^2^ statistic. Sensitivity analysis and subgroup analysis will be used to observe changes in the pooled effect size and heterogeneity between included studies, to assess the reliability and stability of the pooled results. The funnel plot and Egger's and Begg's tests will be used to judge publication bias, and the trim and fill method will be used to correct the funnel asymmetry caused by publication bias. P < 0.05 will be considered to indicate a statistically significant result.

**Results:**

This systematic review and meta-analysis will assess the efficacy of ACE inhibitors versus ARBs on IR in hypertensive patients.

**Conclusions:**

Our study will show the efficacy of ACE inhibitors versus ARBs on IR in hypertensive patients. And it may find a more beneficial therapeutic option to assist clinicians in making clinical decisions.

**Ethics and dissemination:**

This study is a protocol for systematic review and meta-analysis of the efficacy of ACE inhibitors and ARBs on IR in hypertensive patients. This systematic review and meta-analysis will be published in a journal and disseminated in print by peer-review.

**INPLASY registration number:**

INPLASY202050032.

## 1 Introduction

Insulin resistance (IR) is an abnormal condition in which the body responds to both endogenous and exogenous insulin. IR is considered as the common core pathological basis of metabolic disorders and plays an important role in the occurrence of various cardiovascular and cerebrovascular diseases based on atherosclerosis.^[1–3]^ Patients with essential hypertension are resistant to insulin-mediated glucose uptake. Hyperinsulinemia may also lead to atherosclerosis and promote vascular smooth muscle proliferation and hypertrophy by locally activating the renin-angiotensin-aldosterone system (RAAS).^[4]^ It is well known that hypertension is one of the most prevalent cardiovascular risk factors. And hypertensive patients with high IR have a higher incidence of cardiovascular and cerebrovascular events.^[5]^ Moreover, people with hypertension have a high prevalence of IR and are of relatively higher risk in developing type 2 diabetes mellitus.^[6,7]^ Indeed, the management and treatment costs of a hypertensive patient with diabetes are far greater compared with a nondiabetic patient. Therefore, when selecting antihypertensive agents for hypertensive patients, not only the antihypertensive effect but also effects on insulin sensitivity, glucose metabolism, and lipid metabolism should be considered.

Large epidemiological studies have proved that the renin-angiotensin system (RAS) was closely related to glucose homeostasis.^[8]^ Moreover, many studies have identified antihypertensive drugs that act by intervening in the RAS as overall having beneficial effects on glucose homeostasis.^[9]^ And a meta-analysis of 10 studies (n = 76,000) further proved this.^[10]^ Animal model-based studies also found that treatment with ACE inhibitors or ARBs induces metabolic improvements in conditions of IR.^[11,12]^ Besides, numerous studies have suggested that IR could be important in the pathogenesis and progression of hypertension.^[13,14]^

ACE inhibitors and ARBs are 2 types of the RAS blockers commonly used because of their remarkable effects in decreasing blood pressure and having no adverse effect on glucose and lipid metabolism. But comparing with ARBs, ACE inhibitors are limited by the typical adverse drug reaction of dry cough in clinical. Angiotensin 2 interaction with angiotensin II type I receptor (AT1R) leads to vasoconstriction, cell growth, inflammation, fibrosis, and oxidative stress.^[15]^ AT1R blockade is likely to reduce these adverse effects, which may be the possible mechanism that ARBs can improve IR. The beneficial effects of ACE inhibitors may rely on a higher angiotensin-converting enzyme 2/angiotensin-converting enzyme (ACE2/ACE) ratio and reduce oxidative stress and glucotoxicity by improving glucose-stimulated insulin secretion and islet function while reducing oxidative stress and inflammation.^[16]^ In recent years, a series of randomized clinical trials (RCTs) have compared the efficacy of ACE inhibitors with ARBs in improving IR. However, the results of these RCTs remain controversial. A systematic review and meta-analysis will be appropriate to resolve the current controversy and reach a conclusive result.

Thus, this systematic review and meta-analysis will aim to evaluate the effect of ACE inhibitors versus ARBs on IR in hypertensive patients. And such a study may find a more beneficial therapeutic option, that of reducing target organ damage.

## 2 Materials and methods

The current systematic review and meta-analysis will be reported following the Preferred Reporting Items for Systematic Reviews and Meta-Analyses (PRISMA).^[17]^ This review protocol is registered in the INPLASY register (registration number: INPLASY202050032).

### 2.1 Search strategy

**Figure 1:**
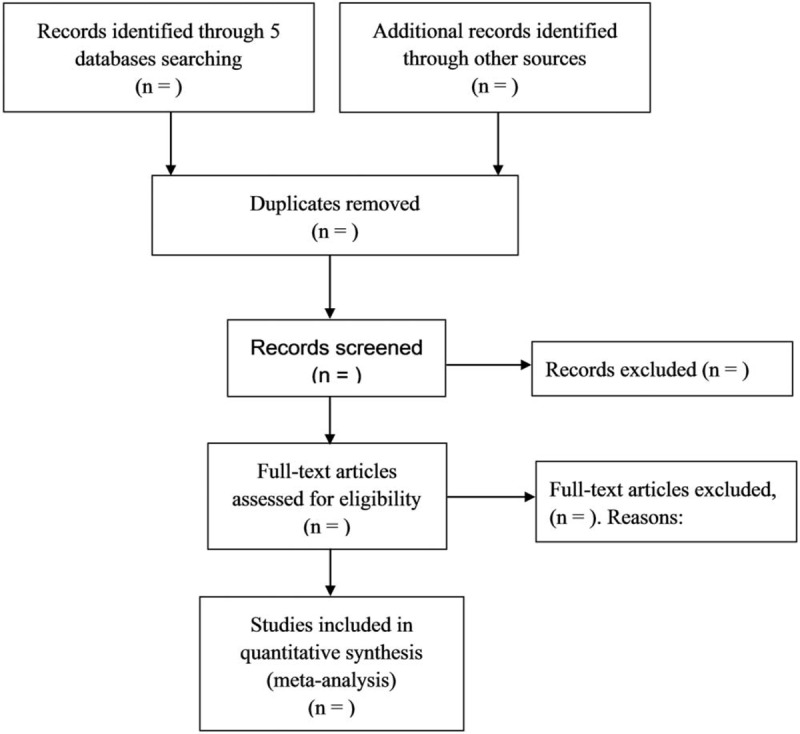
Flow diagram of study selection.

Databases including Medline, the Cochrane Library, EMBASE, Web of Science, and Cochrane Central Register of Controlled Trials (CENTRAL) will be searched. According to the PICOS principle, the search strategy will include the following terms: ((“hyperinsulinemic euglycemia clamp” or “euglycemic clamp” or “glucose clamp” or “HOMA”or “HOMA-IR” or “homeostasis model assessment” or “QUICKI” or “minimal model analysis” or “minimal model” or “insulin resistance” or “IR” or “insulin sensitivity” or “index of insulin sensitivity” or “glucose” or “insulin”) AND (“angiotensin converting enzyme inhibitor” or “angiotensin converting enzyme inhibitors” or “ACEI” or “ACE inhibitor” or “ACE inhibitors” or “ACEIs” or “alacepril” or “benazepril” or “captopril” or “ceranapril” or “cilazapril” or “cilazaprilat” or “delapril” or “enalapril” or “fosinopril” or “fosinoprilic acid” or “imidapril” or “libenzapril” or “quinaprilat” or “ramipril” or “ramiprilat” or “rentiapril” or “saralasin” or “spirapril” or “temocapril hydrochloride” or “teprotide” or “trandolapril” or “zofenopril”) AND (“angiotensin recept” or “antagonists” or “angiotensin II type 1 receptor blocker” or “angiotensin II type 1 receptor blockers” or “angiotensin receptor blocker” or “angiotensin receptor blockers” or “angiotensin II receptor blocker” or “angiotensin II receptor blockers” or “ARB” or “ARBs” or “losartan” or “cozaar” or “valsartan” or “diovan” or “telmisartan” or “micardis” or “candesartan” or “tasosartan” or “verdia” or “eprosartan” or “irbesartan”)). The ClinicalTrials.gov registry will be also searched for unpublished trials and authors will be contacted for additional information if necessary. Relevant references from included studies will be sought to retrieve additional eligible studies. No limits will be set on language, publication date, and type. Flow diagram of study selection will be in Figure 1.

### 2.2 Inclusion and exclusion criteria

The inclusion criteria will be as follows: RCTs with any follow-up duration and sample size; participants studied have a diagnosis of essential hypertension; the experimental group will be given ACE inhibitors, and the control group will be given ARBs; reported quantitative outcomes: the primary outcome will be IR (using recognized methods such as homeostasis model assessment of insulin resistance, glucose infusion rate, Quantitative Insulin-Sensitivity Check Inde), secondary outcomes will be blood pressure, fasting plasma glucose, and fasting plasma insulin. The exclusion criteria will be as follows: non-RCTs; reports lacking relevant or sufficient data; duplicate literature.

### 2.3 Data extraction and management

Relevant data extraction will be performed by 2 researchers (JY and XYG) independently, and the third researcher (XYS) will be involved in a discussion for any disagreements. The following information of eligible articles will be extracted to a prepared data extraction form: author, year of publication, country of origin of the population studied, study design, sample size, duration, health status, mean age, number of males, drug doses, intervention, and outcomes. If raw data will not be directly provided in the text or tables, figures in the study would be referred to. Once relevant details will be insufficiently reported in studies, authors will be contacted by e-mails and the ClinicalTrials.gov register will be searched for further information.

### 2.4 Quality assessment

According to the Cochrane collaboration's update tool for assessing the risk of bias (Version 5.1.0),^[18]^ 2 reviewers (JY and XYG) will assess the quality of the studies independently, and the third researcher (SMF) will be consulted for any disagreements. The risk of bias will be classified as low, unclear, or high risk by evaluating the 7 components as random sequence generation, allocation concealment, blinding of outcome assessment, blinding of participants and personnel, incomplete outcome data, selective outcome reporting, and other bias. If necessary, we will try to e-mail the authors for extra information.

### 2.5 Statistical analysis

Stata 12.0 software will be used for statistical analysis. Dichotomous data will be expressed as the odds ratio (OR) with a 95% confidence interval (CI), and continuous data will be presented as the standardized mean difference with 95% CI. *P* < .05 will be considered to indicate a statistically significant result.

### 2.6 Units of analysis issues

All parallel-designed studies will be included in this review. For cross-over trials, only the 1st treatment period data will be analyzed. For studies with multiple control groups, the unit of analysis will be used to each of all control groups.

### 2.7 Dealing with missing data

For insufficient or missing data, we will contact the authors by e-mail or phone as much as possible. Where data are unobtainable, we will assume that an event, without a reported outcome, has not occurred in participants and we will analyze only the available data. All analyses will be performed based on the intent-to-treat principle.

### 2.8 Assessment of heterogeneity

Heterogeneity will be tested by χ^2^-based Cochran Q statistic (*P* < .10 indicated statistically significant heterogeneity) and *I*^2^ statistic. If *I*^2^ < 50% and *P* > .1, a fixed-effects model will be used to pool the estimations across studies. If *I*^2^ ≥50% or *P* ≤ .1, after excluding clinical heterogeneity between studies, the random-effects model will be used.

### 2.9 Data synthesis

If there are sufficient studies and comparable outcomes, we will perform a meta-analysis. If not, we will perform a systematic review.

### 2.10 Subgroup analysis and investigation of heterogeneity

Subgroup analysis will be performed to explore the differences in the methodologic quality, race/ethnicity, sample size, and duration.

### 2.11 Sensitivity analysis

Sensitivity analysis will be used to observe changes in the pooled effect size and heterogeneity between included studies, to assess the reliability and stability of the pooled results.

### 2.12 Assessment of reporting biases

The funnel plot and Egger's and Begg's tests will be used to judge publication bias, and the trim and fill method will be used to correct the funnel asymmetry caused by publication bias.

### 2.13 Confidence in cumulative evidence

In this study, the level of evidence on all outcomes will be appraised by using an approach based on the Grading of Recommendations Assessment, Development, and Evaluation (GRADE). The quality of the body of evidence will be assessed based on 5 factors, including study limitations, effect consistency, imprecision, indirectness, and publication bias. The assessments will be categorized as high, moderate, low, and very low quality.

## 3 Discussion

The close relationship between RAS and IR is not a recent observation. Increased expression of the RAS components and high expression of local RAS elements damage the insulin signaling cascade and contribute to both IR and type 2 diabetes mellitus onset.^[19]^ RAS also has multiple effects in the central nervous system, skeletal muscle, liver, and adipose tissue that may interfere with insulin action. Studies have shown that ACE inhibitors and ARBs can potentially improve insulin resistance in hypertensive patients compared with other antihypertensive drugs.^[20]^ Furthermore, to date, some RCTs have compared ACE inhibitors with ARBs on the efficacy of improving insulin resistance; however, the results are not inconsistent. On this basis, we will summarize the available evidence to compare ACE inhibitors with ARBs on the effect of insulin resistance in hypertensive patients. And such a study may find a more beneficial therapeutic option for hypertensive patients with IR and assist clinicians and health professionals make clinical decisions.

## Author contributions

**Data analysis:** Xiaoyan Shi, Simin Fan.

**Data extraction:** Jia Yao, Xiayu Gong.

**Funding acquisition:** Qiu Chen.

**Methodology:** Qiu Chen.

**Project administration:** Qiu Chen.

**Resources:** Qiu Chen.

**Software:** Junmin Chen.

**Writing – original draft:** Jia Yao, Xiayu Gong.

**Writing – review & editing:** Jia Yao, Xiayu Gong.
